# Mainstreaming of biomedical waste management: Best practices for clinical laboratories in Africa

**DOI:** 10.4102/ajlm.v14i1.2881

**Published:** 2025-07-31

**Authors:** Pasipanodya I. Machingura Ruredzo, Bettina Chale-Matsau

**Affiliations:** 1 Department of Laboratory Diagnostic and Investigative Sciences, Faculty of Medicine and Health Sciences, University of Zimbabwe, Harare, Zimbabwe; 2 Department of Chemical Pathology, Faculty of Health Sciences, University of Pretoria, Pretoria, South Africa; 3 National Health Laboratory Service, Pretoria, South Africa

## Introduction

Biomedical waste comprises liquid and solid material generated during provision of healthcare services encompassing diagnostics and patient management. As these toxic, radioactive, or infectious products are potentially hazardous to human and environmental health, proper management of biomedical waste is of concern.^1^ Modes of exposure to biomedical risks include cuts or pricks, intimate contact with skin or mucous membranes, inhalation or ingestion of hazardous substances.^2^

The World Health Organization reports that an estimated 15% of waste generated by healthcare activities is hazardous material which may be infectious, toxic, carcinogenic, flammable, corrosive, explosive, or radioactive.^3^ The open burning and low temperature incineration of such waste may cause emission of dioxins, furans, and particulate matter.^4,5^ As patient care is mostly associated with submission of samples (e.g. fluid or biopsy specimens) to a pathology laboratory to aid in diagnosis, clinical laboratories are among the major sources of healthcare waste. In low- and middle-income countries (LMICs), healthcare waste is often not separated into hazardous or non-hazardous wastes, causing the quantity of hazardous waste to be much higher.^3,6^ The objective of this editorial is to highlight the health and environmental risks associated with biomedical waste management in clinical laboratories and to point out reasonable measures to best address these challenges.

## Medical laboratory waste

Medical laboratory wastes carry a higher potential for infection and cause of injury by accidental needle sticks. It is reported in some countries that hazardous medical waste is handled and disposed together with household waste, which causes a health risk to municipal works, the public, and the environment.^7^ Ideally, the management of the laboratory waste that includes biological agents should include a validated decontamination determined by the risk assessment process as stipulated in international and local waste disposal guidelines. The decontamination process can be conducted by a combination of chemical disinfectants and autoclaving, and incineration can be used to supplement in some cases. Thus, it is essential that laboratory personnel have basic knowledge on cleaning, disinfection, and sterilisation.^3^

## Laboratory waste management status quo in low- and middle-income countries

Healthcare services in LMICs continue to grow, and increased availability and use of diagnostic services is leading to growing rates of biomedical waste generation. Large quantities of biomedical waste are generated in medical laboratories. Most of these originate from wards, clinics, and surgical theatres as fluid (e.g. blood, urine, saliva, cerebrospinal fluid) and tissue obtained from patients during processes associated with diagnosis and treatment monitoring.^8,9^

Waste in the laboratory is handled by medical laboratory professionals, who in some cases have limited training in waste management. There are items that are not harmful, such as packaging, but that are often collected and stored with hazardous material. Because of a lack of training in waste management, medical laboratory professionals have limited knowledge of proper waste segregation. Most clinical laboratories entrust the responsibility of waste removal and disposal to a registered service provider, with the expectation that proper measures are followed. Following pick-up, biomedical waste is taken to disposal sites and manually sorted prior being subjected to respective mechanisms that include decontamination prior to being taken for recycling, landfills, or incineration, the products of which are then disposed in landfills. Because of resource restraints in LMICs, waste handlers may not have adequate protective clothing or training in waste management. In many instances, landfills are shallow and incineration is conducted in open facilities at suboptimal temperatures,^10^ further worsening the hazardous nature of biomedical waste.

## Causes of poor waste management

Poor waste management is caused by limited legal frameworks, lack of awareness of the health hazards of the waste, inadequate training in appropriate waste management, lack of proper waste management and disposal systems, inadequate financial and human resources, and lack of prioritisation of waste management.^11^ Furthermore, many countries lack appropriate regulations, or do not monitor and enforce them.^3^ The prevailing unpleasant economic climate in most LMICs further compromises biomedical waste management, thus pushing the issue further down on the agenda of policymakers. It is important even when biomedical waste management policies are available to renew them periodically, in order to incorporate new research and system development points.^2^ The World Health Organization provides material that can be utilised to develop both standard and more specific procedures for dealing with pathogenic agents in the laboratory.^3,12^

## Potential health and environmental impact of improper waste management

Healthcare activities generate different kinds of waste from non-hazardous to hazardous. While harmful waste constitutes a small fraction of total waste, pooling all biomedical waste together increases the amount that must be disposed of. Hospitals and laboratories in LMICs lack appropriate waste segregation, storage, collection, and disposal practices, increasing the health risk to health professionals, waste handlers, and the rest of the community.^13,14^ Potential harms include exposure to infectious pathogen-bearing waste material, toxins, and carcinogenic chemicals that may be inhaled, absorbed through skin, or introduced to the body from needle sticks.^13^

Certain medical waste such as noxious chemicals and persistent spores from pathogens may remain airborne for an extended period of time. Open burning and incinerators produce outdoor air pollution. Products of partially incinerated waste disposed of in low-lying fields can easily seep into water systems. Biological, chemical, and radioactive materials all have the potential to contaminate water bodies.^4^ Bacteria in the waste has the potential to infiltrate ground water and surface water. Liquid laboratory waste from cleaning work benches and glassware and effluent from analysers are incorporated into municipal wastewater. Municipal wastewater is partially treated and released into rivers. This inappropriate handling of biomedical waste can have long-lasting effects on humans, animals, and plants, affecting the food chain and future generations. Regulated, sustainable waste management practices can reduce the harmful effects of biomedical wastes.^7^

## Tackling poor waste management

Pre-service training and refresher courses for laboratory personnel must encompass this important aspect of biosafety. Studies have shown that 85% of laboratory waste is non-harmful and is generally combined with the 15% of hazardous waste that is harmful, thus materially increasing the load of potentially harmful waste.^3^ Proper segregation of waste and appropriate disposal is required to safeguard human health and minimise environmental impact.^8^ Laboratory personnel require skills training in inventory management, including regular checking of expiration dates and avoiding overstocking.

Training institutions must continually update their training on safety to ensure appropriate pre-service training on laboratory waste management and support laboratories though continuing professional development on biosafety. Laboratory personnel must take due consideration of the safety of themselves and the population at large, with governments taking leadership to ensure there are appropriate regulations, monitoring, and enforcement to ensure compliance.^2^

Laboratory personnel have an ethical obligation to ensure the safety of the laboratory and community at large. Laboratory waste audits can be conducted to guide planning and decision-making for laboratories. Such audits can provide data on the amount, type, and laboratory areas where waste is generated to provide a baseline for waste management initiatives.^11^ Laboratory management personnel are expected to adhere to and encourage implementation of national and international guidelines for biomedical waste management, and to ensure that laboratory personnel are familiar with these regulatory frameworks.^15^

## Conclusion

Research in waste management is also essential to help provide evidence for policy review and strengthening. Clinical laboratories may benefit from conducting audits that will provide perspective on the quantities of each category of waste generated, so as to streamline management strategies. In order to realise improvements in biomedical waste management, laboratories should consider inclusion of training and regular refresher courses for medical laboratory professionals that can be incentivised as part of continuous professional development. This is a call for laboratory professionals to keep waste management on the radar as they practise.
